# Investigation of feet functions of large ruminants with a decoupled model of equivalent mechanism

**DOI:** 10.1242/bio.023630

**Published:** 2017-04-15

**Authors:** Qun Zhang, Kun Xu, Xilun Ding

**Affiliations:** 1 Space Robot Laboratory, School of Mechanical Engineering and Automation, Beihang University, Beijing 100191, China; 2 State Key Laboratory of Robotics and System, Harbin Institute of Technology, Harbin 150001, China

**Keywords:** Ruminant foot, Ligament, Adaptation, Kinematics, Energy storage

## Abstract

Cloven hooves of ruminants adapt to diverse terrain, provide propulsive force and support the whole body during movement in natural environments. To reveal how the feet ensure terrain adaptability by choosing the proper configurations and terrain conditions, we model the feet of ruminants as an equivalent mechanism with flexion-extension and lateral movement decoupled. The upper part of the equivalent mechanism can flex and extend, while the lower part performs the lateral movement. Combination of the two parts can adapt to longitudinal slope (anterior-posterior) and transverse slope (medial-lateral), respectively. When one of two digits closes laterally, the workspace of the other decreases. The distal interdigital ligament between two digits limits their motion by elastic force and stores energy during movement. Differences in elastic energy variation of the ligament on different transverse slopes are characterized based on the configurations of two digits and the elastic energy between them. If the upper one of two symmetric digits is fixed, the foot landing on the grade surface (2°) shows greater capacity for absorbing energy; otherwise, level ground is the best choice for ruminants. As for the asymmetric digits, longer lateral digits enhance the optimal adaptive lateral angle. The asymmetry predisposes the feet to damage on the hard ground, which indicates soft ground is more suitable.

## Abstract (precis)

**Summary:** This study shows that level and soft grounds are best for symmetric and asymmetric digits, respectively, as revealed by kinematic analysis using a decoupled equivalent mechanism of the foot of ruminants.

## INTRODUCTION

As a result of natural selection, the ungulate herbivores such as horse, cattle and goat are specialized to bear a large amount of poorly digestible food and to maintain long-distance continuous movement (König and Liebich, 2004); ruminants, which have two main digits, are one of the most remarkable representatives and are widely distributed around the world (Maglio and Cooke, 1978). Despite the diverse living environment, ruminants have feet (the most distal parts) of similar structure to cattle, camel, goat, deer, etc. (Sisson, 1921; Shen, 2008; Keller et al., 2009). With simple, reliable and strong feet, they adjust very well to the terrain on which they feed, mate and avoid predators; for example, cattle adapt to the soft ground (Chen et al., 2007; Flower et al., 2007; Hernandez-Mendo et al., 2007) and camels have special, soft, relatively big feet to cross deserts (Wang et al., 1995). In order to feed on grass, shrub or trees, goats and blue sheep are able to climb up and down cliffs and ledges solidly and fleetly (Zhang, 2011; Luo, 2011), and while contacting the ground, their cloven hooves can spread apart and ‘grasp’ the rock to avoid slipping (Manning et al., 1990; Brandborg, 1955). Previous studies (Carroll et al., 2008; Lee et al., 2008; Arnold et al., 2013; Carvalho et al., 2007) consider the feet as a whole to discuss the moving characteristics because feet are thought to contribute little to the forward progression (Fischer and Blickhan, 2006); however, this view may underrate the importance of feet. ‘Forward progression’ is not the sole utility of the feet. The feet are ingenious, constituted by skeleton, multiple joints, ligaments, muscles, subcutis and some skin modifications (König and Liebich, 2004).

Also, tendons and ligaments, as the main elastic components of mammals, have been widely examined. Tendons and ligaments constitute the passive support mechanism to reduce muscle and skeleton fatigue by carrying tension to mitigate the bending moment in bones (Ananthanarayanan et al., 2012). Among them, the principal springs are tendons in the leg and ligaments in the foot (Alexander, 1988). The spring-mass model is applied to explain the high speed of animals during running and hopping (Alexander, 1990; Blickhan, 1989). The long distal tendons and suspensory ligaments provide great compliance in distal joints to mitigate the impact force and to dampen the high-frequency vibration (Wilson et al., 2001). In addition, the tendons take tensions to lessen the bending stress at bone (Ananthanarayanan et al., 2012). Owing to these structures, MCP (metacarpophalangeal joint) and MTP (metatarsophalangeal joint) of goats act as the principal springs of the legs and do significant work during level, uphill and downhill running by flexing and extending (Lee et al., 2008). In addition, intracapsular, capsular or extracapsular joint ligaments strengthen the joints and limit the joints to the normal rotation range of motion (Frank, 2004). Alexander and Bennett (1987) summarized several functions of ligaments in hinge joints, elaborating on the important functions of maintaining the integrity of the joints, limiting and controlling joint movement. However, the extent to which the feet of ruminants with two main digits contribute to adapting different terrain types was not investigated, and how the ligaments between two digits limit the movement of two digits is still unclear.

The claws of dairy cattle have caught much attention due to the increasing claw disorders and lameness in artificial housing environments (Murray et al., 1996; Cramer et al., 2008). The present study investigated the digits of artiodactyls by measuring the length of the phalanges. It was suggested that length asymmetry between the lateral and medial digit (Nuss and Paulus, 2006; Muggli et al., 2011) may cause the lameness of domestic cattle housed on hard surfaces. Besides, the wild ruminants have an uneven length of digits (Keller et al., 2009). Ground reaction forces (GRF) during standing and walking indicated overload of the lateral hind claws which was deemed to be the main inducing factor for lameness (Van der Tol et al., 2003).

In the current study, we utilize a decoupled model to analyze the movement of two digits for clarifying the limitation and energy storage of the distal interdigital ligament. By introducing the length asymmetry of the digits, the terrain adaptability of the ruminants is analyzed.

## RESULTS

**Fig. 1.**
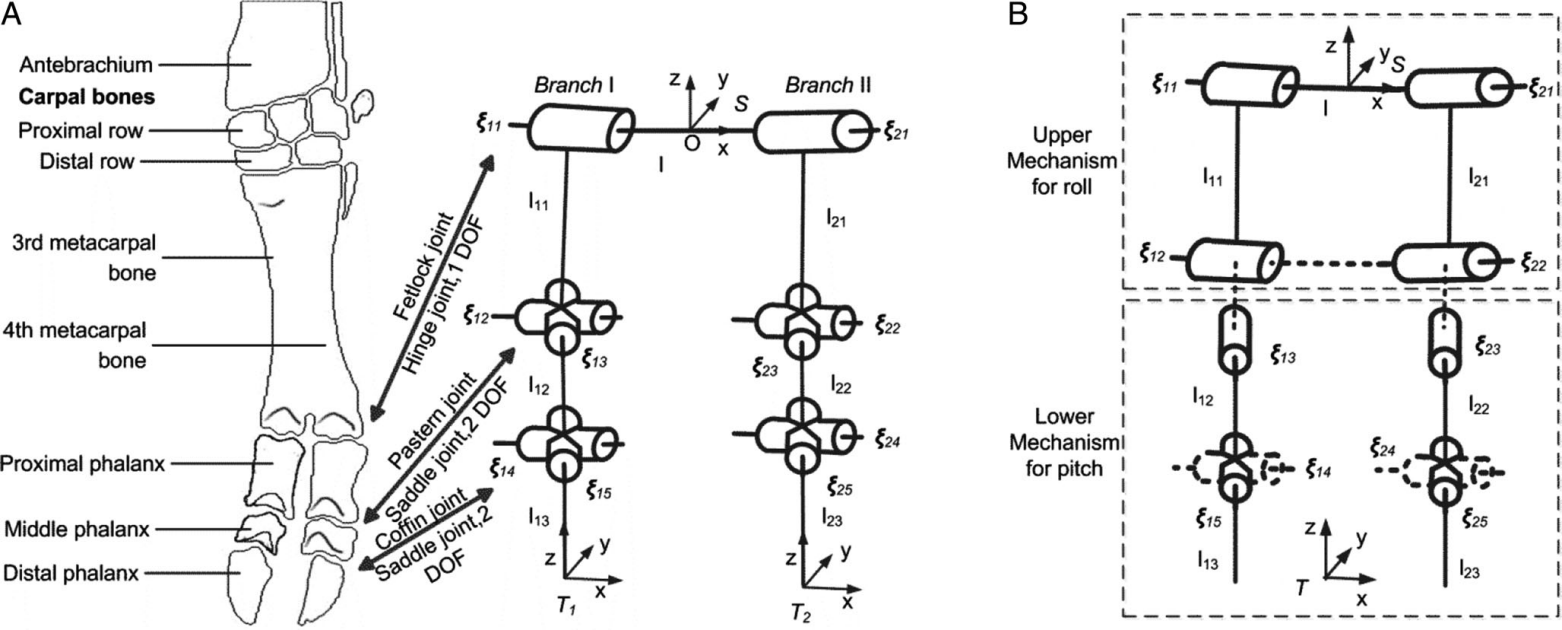
**Equivalent mechanisms of the foot of ruminants.** (A) The skeleton and articulation of the ox's manus (schematic), whose digits can be equivalent to an articulated mechanism. (B) Joint ***ξ_14_*** and ***ξ_24_*** are fixed in the decoupled mechanism, flexions and extensions and lateral movements are performed in the upper mechanism and lower mechanism, respectively.

**Fig. 2.**
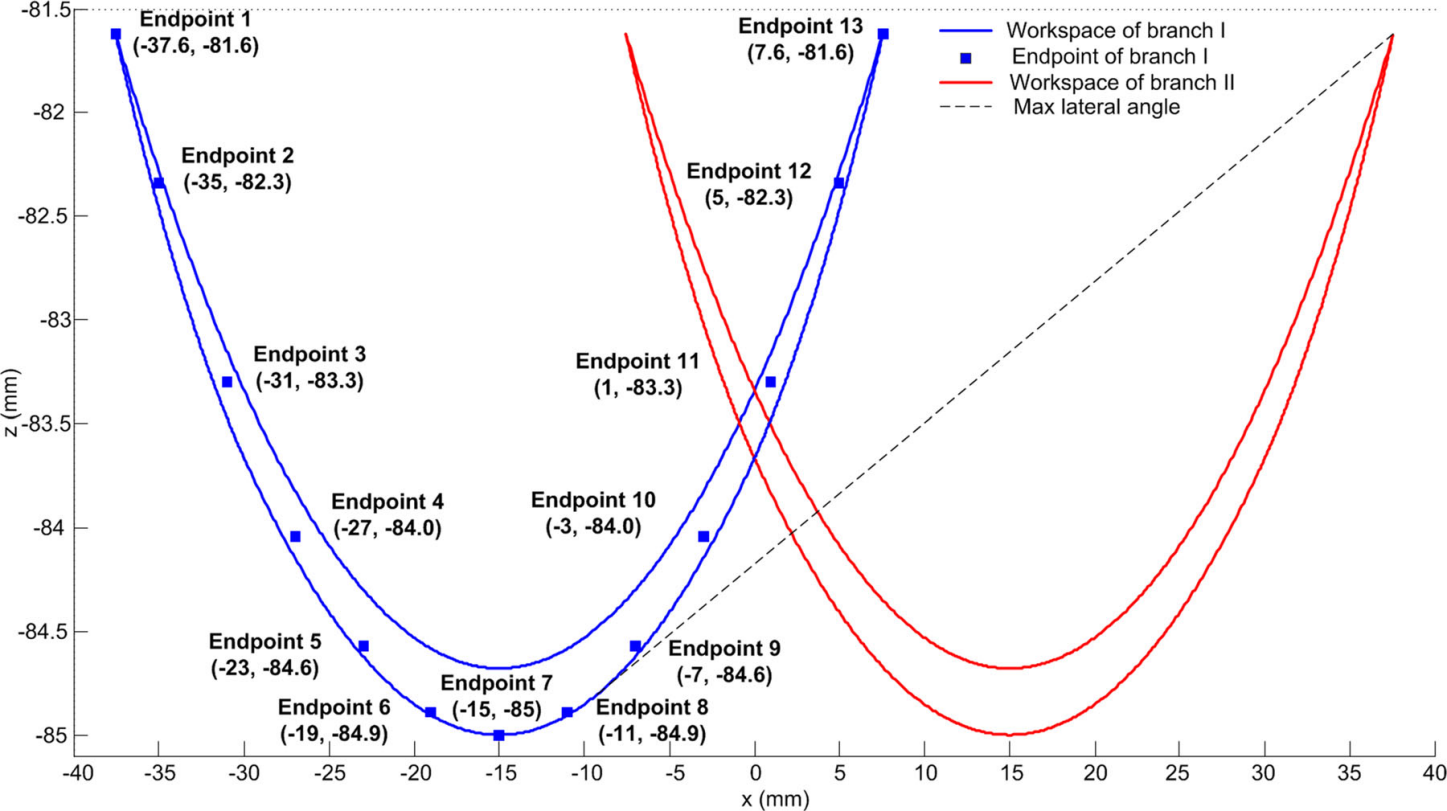
**Workspace of the endpoints of two symmetric branches with angle limit and no link interference.** The solid squares indicate the positions of the endpoint of Branch I. 13 positions are chosen within the workspace of Branch I, numbered from 1 to 13. The dashed line denotes the maximal lateral slope angle that the feet can adapt.

**Fig. 3.**
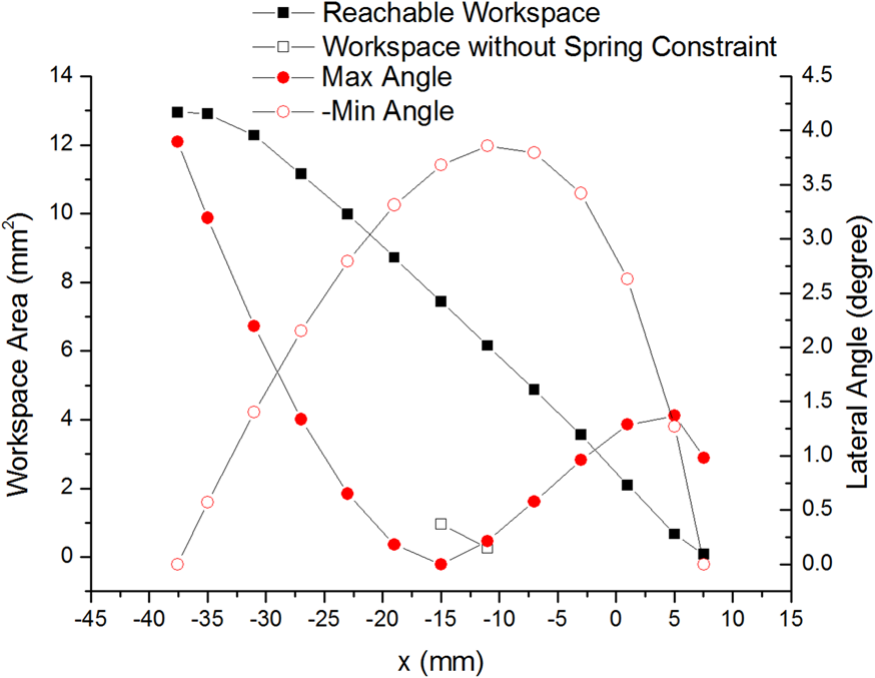
**Workspace area of Branch II and lateral slope angle versus the *x* coordinate of the endpoint of Branch I (symmetric digits).** The black solid squares represent the reachable workspace of Branch II given the configuration of Branch I. The black open squares illuminate the area of workspace without the elastic constraint (the ropes are all relaxed). Within the workspace of Branch II, only two configurations of Branch I lead to the small unrestricted workspace. The red solid circles are the maximal lateral angle that Branch II can reach, while the red open circles are the opposite of the minimal lateral angle.

With the simple decoupled model (the lower mechanism) in Fig. 1B, the available workspace of the endpoints of two symmetric branches which perform the left and right rotation (abduction and adduction) is determined as shown in Fig. 2. Considering the angular range of joints in Table 1, the area of the workspace is determined using the polar coordinates search method mentioned in Materials and methods. The workspace of two branches overlaps in the middle, but it is impossible for two endpoints to reach the intersection area concurrently owing to the link interference. Thus, the lateral angle reaches the maximum as shown in Fig. 2, which is 3.89°, much less than 20° (the maximal pitch angle of one digit). Fig. 3 shows how the workspace of two branches interacts between each other. When the configuration of Branch I is determined, the workspace of Branch II cannot reach the whole workspace in most cases. With the link interference and angular limitation, the workspace of Branch II decreases as Branch I moves right (the *x* coordinate increases). When Branch I moves to the extreme left positon (*θ*_13_=10°, *θ*_15_=10°), Branch II can reach the whole workspace as shown in Fig. 2. When Branch I moves to the extreme right (*θ*_13_=–10°, *θ*_15_=–10°), the area of the available workspace is smallest.

**Table 1.**
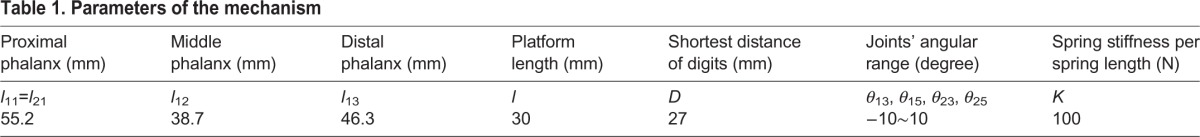
**Parameters of the mechanism**

Fig. 3 shows the free length of the ropes dividing the available workspace of Branch II into two parts at some certain configurations of Branch I (e.g. *θ*_13_=0, *θ*_15_=0): the workspace without elastic constraint (free workspace) and the workspace with elastic constraint. In the free workspace, Branch II can move impervious to Branch I, as there is no elastic force between two branches. In contrast, Branch II tends to return to the configuration of smaller elastic force or elastic energy in the workspace with elastic constraint. Nevertheless, the free workspace emerges only when the configurations of two branches are near the reference configuration and is a small proportion of the whole reachable workspace (12.8% at the 7th configuration, 4.06% at the 8th configuration). Except at these certain configurations, the Branch II is restricted by the springs in most cases.

The lateral slope angle also changes while Branch I moves right. Negative angles represent Branch II on the upper position of the slope, while positive angles mean that Branch II is on the lower position of the same slope. With Branch I moving right, the maximal absolute angle decreases first (from 3.89°, Branch I on the upper place) and then increases, and it reaches the extremum (3.86°, Branch I on the lower place). After then, analogous to the workspace area, it decreases and becomes smallest (0.98°) when Branch I reaches the extreme right.

**Fig. 4.**
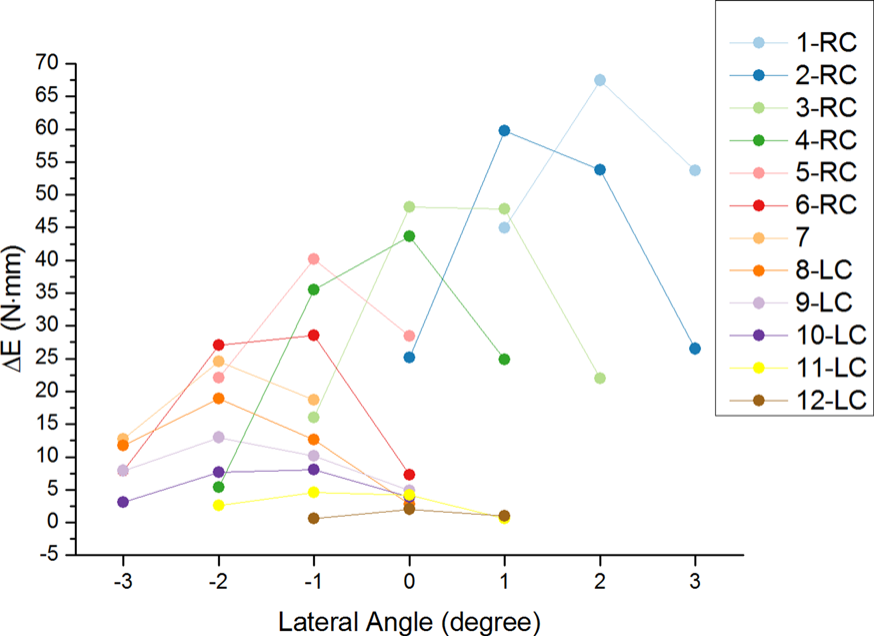
**The elastic energy variation versus the lateral slope angle at different configurations of Branch I (symmetric digits).** The line number corresponds to the endpoint positions in Fig. 2. LC indicates the configuration of Branch I is left configuration, while RC means right configuration.

**Fig. 5.**
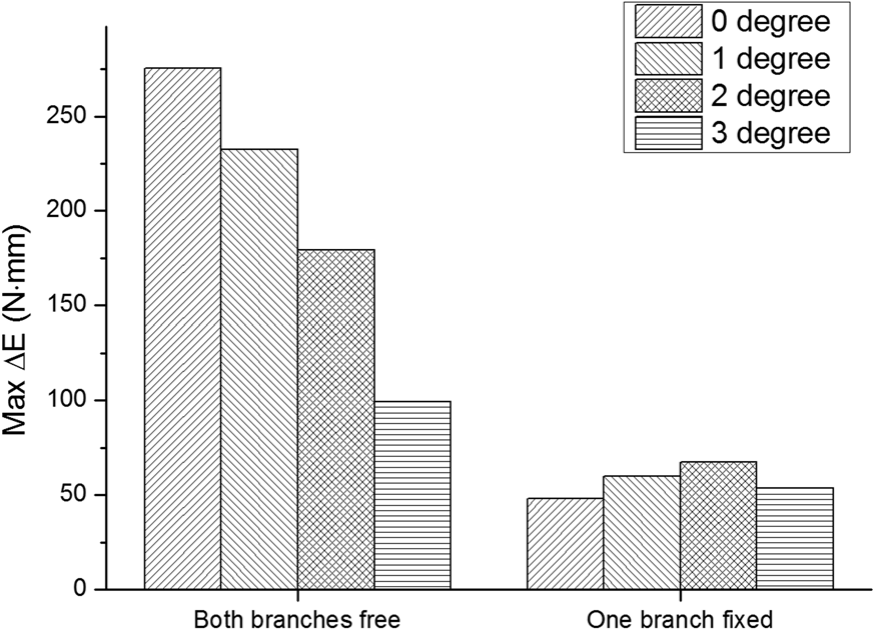
**The maximum elastic energy variation of two patterns at the lateral angles of 0, 1, 2, 3° (symmetric digits).** One pattern is that one of two branches is fixed; the other pattern is that both branches are free to move. Branch I moves between the 13 configurations.

When two branches are on a slope and the configuration of Branch I remains unchanged, no relative motion exists between the base and the slope. In most cases, as the endpoint of Branch II moves away from the Branch I, the elastic energy increases (Fig. 4). Δ*E* reaches its peak value at the medium lateral slope angle within its range and is larger when the endpoint of Branch I is at the left part of the workspace (configuration 1-4 in Fig. 2 and Branch I locates on the upper). Similar to the workspace area in Fig. 3, Δ*E* decreases along with Branch I moving right (Fig. 4). If both branches are allowed to move during stance phase, Δ*E* is much larger and more sensitive to the lateral angle (Fig. 5). Δ*E* peaks during the level slope.

**Fig. 6.**
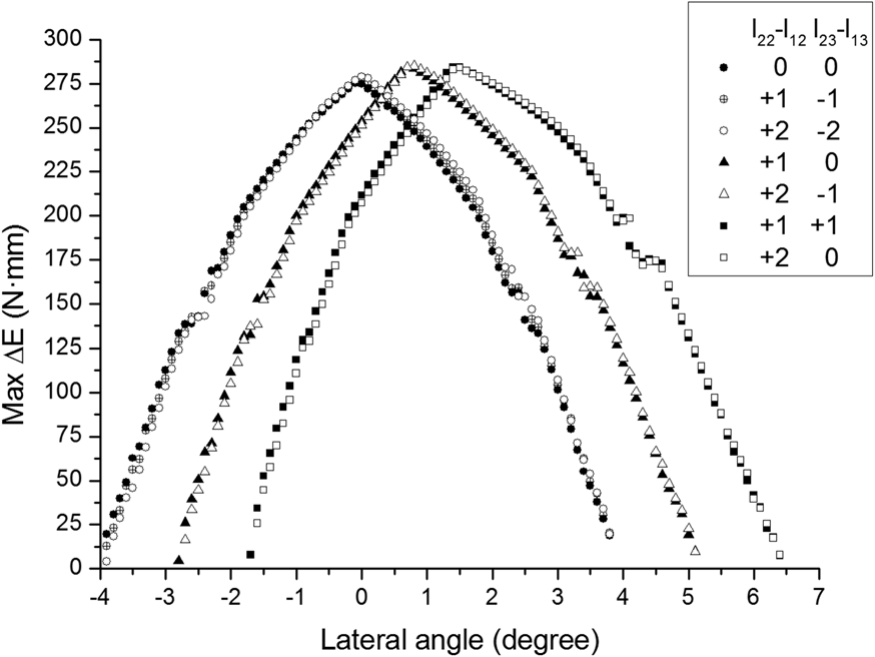
**The maximum elastic energy variation of asymmetric and symmetric branches against lateral slope angle.** Both branches are free to move, and energy variation is calculated every 0.1° of lateral slope angle. The *x* coordinate of points indicates the right boundary of 0.1° interval.

Fig. 6 shows the maximum elastic energy storage from asymmetric branches compared with symmetric ones. The small increase of the overall length of Branch II performs little influence on the curve shape and the peak value, but makes the peak value shift rightwards. The energy variation does not differ a lot with the relative length between phalanges. It peaks at 0° of lateral angle with equal branches, 0.8° with 1 mm longer branch, 1.5° with 2 mm longer branch.

## DISCUSSION

In this study, a decoupled model and method that can be utilized to investigate the functions of the feet of large ruminants with the similar foot structure is presented. Some constraints are added to the model in Fig. 1A to decouple the movement of the feet. Owing to the decoupled model, the movement of the feet is explicit. The upper mechanism performs anterior-posterior movement, while the lower mechanism performs the lateral movement. The anterior-posterior angle and lateral angle do not interrelate with each other. Hence the feet are easy to control, and the unexpected movements are avoided.

The upper mechanism is comprised of two fetlock joints (flexion and extension) and two pastern joints (restricted to flexion and extension), which can only flex and extend in the sagittal plane. The fetlock joints and pastern joints in two digits flex and extend synchronously. Thus the movement is similar to that of one digit feet, for example in horse, except that two digits make greater stiffness and better stability. In addition, the axis of movement is parallel to that of other joints of the limb. Thus the upper mechanism extends the limb of ruminants and adds two more equivalent joints. The speed increase is attributed to the elongation of the distal bones (Köhler and Moyà-Solà, 2001), and more flexion joints augment the dexterity of the limb. The functions of the upper mechanism are similar to the corresponding part of horse.

The lower mechanism is responsible for the lateral movement, which is composed of two pastern joints and two coffin joints (all restricted to the lateral movement). By regulating the relative position and the configurations of two digits, the lower mechanism can adapt to the terrain with different lateral slope. The lower mechanism facilitates traversing across a slope with a maximal lateral angle 3.89° with symmetric digits. The phalangeal joints of the horse resemble those of the ruminants, except that the horse has only one digit (König and Liebich, 2004). As a comparison, if we assume the digit of horse has the same angular range, the maximal lateral angle will be 20°. Notably, the structure of two digits diminishes the adaption of the lateral slope. However, ruminants can utilize their digits to provide extra foot-ground adhesion. Their cloven hooves can spread apart when contacting the ground and ‘grasp’ the rock to avoid slipping (Brandborg, 1955; Manning et al., 1990). Even though the tips of the digits are fixed to the ground or the rock (no relative translation), the ruminants have relatively greater dexterity from choosing an appropriately sized rock (Zhang et al., 2015). In conclusion, ruminants sacrifice the dexterity to stabilize the movement.

**Fig. 7.**
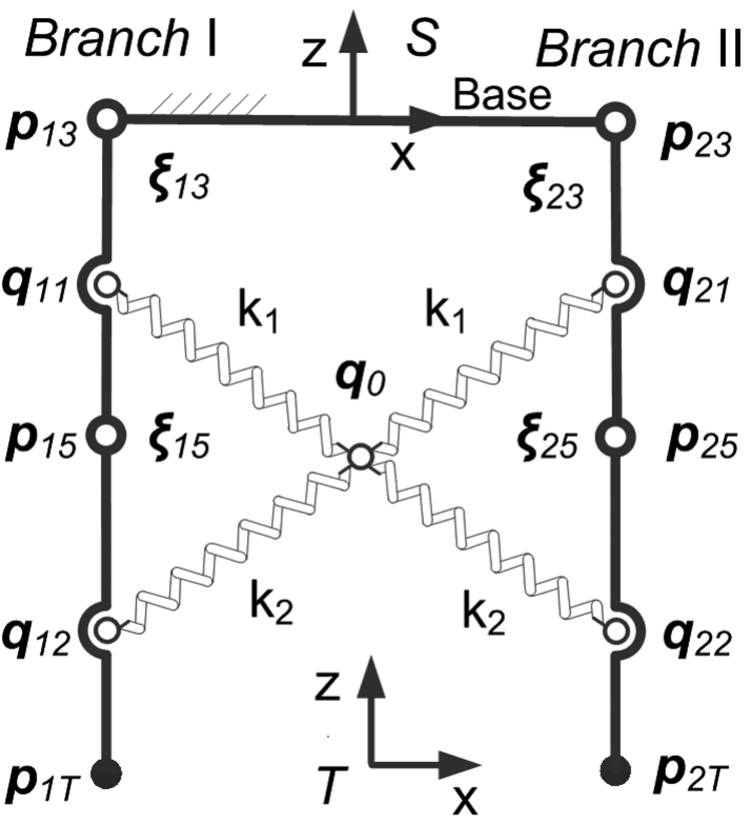
**The equivalent mechanism with the distal interdigital ligament laying to the lower mechanism; the distal interdigital ligament is modeled as four conjoint ropes.**

During stance phase, two digits contacting the ground provides good adhesion and stability. If the configuration of one digit remains unchanged, the distal end of the upper mechanism (the base in Fig. 7) has no relative movement during stance phase. That is, the lateral movement is impervious to the flexion of the limb and is conducive to the movement of animals. Fig. 3 shows that the workspace area of the other digit decreases when the fixed digit moves closer. That is, there will be less workspace for the free digit standing on the slope. As for the lateral angular range, it suffers little effect when the tip of fixed digit is in the left part of its workspace. However, when the fixed digit moves to the extreme right, the angular range becomes very small. Thus, the dexterity of the lower feet depends on the relative configuration of two digits. The fixed one should stay away from the free one to achieve high dexterity. It seems that the digits need to splay out when contacting the ground.

Another function is illuminated as the distal interdigital ligament is added to the decoupled model. Most ligaments span joints and are anchored to the adjacent bones. They are thought to passively stabilize joints and make those joints rotate at an acceptable range when a tensile load is applied (Alexander and Bennett, 1987; Frank, 2004). However, the functions of interdigital ligaments are not intuitional. A relatively simple model (Fig. 7) is utilized in order to probe the functions of the distal interdigital ligament. As discussed above, the configuration of one digit affects the limitation exerted by interdigital ligaments on the other digit. While one digit is subject to the reference configuration, shown in Fig. 7, the other one can move freely near its reference configuration insusceptible to elastic constraint. Hence the lateral movement of joints will be inexpertly unbounded and rapid under the circumstances. It may cause injury to ruminants, as the distal interdigital ligament cannot limit the relative motion of two digits. Fortunately, at most configurations of the digits, the distal interdigital ligament stretches and exerts elastic force.

Some studies refer to the feet for the damping and absorption of energy. The foot pads of most mammals can moderate the maximum force when the foot hits the ground (Alexander, 1990). The digital flexor muscles are mainly used to damp high-frequency oscillations of the limb (Wilson et al., 2001). Between the foot pad and digital flexor tendon, interdigital ligaments play an effective role in the feet of ruminants. A foot landing on the ground will provide a force to support and propel the locomotion of animals. Under the GRF, the springs in the feet will deform to generate the elastic force. If the elastic energy exceeds the capacity of a foot, the springs of the foot may be torn and the joints may be damaged. The distal interdigital ligament, as a cruciate ligament between the main digits of ruminants, is stretched when two digits spread out, limiting the opening angle. Given the configuration of two digits, the variation of elastic energy indicates energy storage/absorption.

If ruminants can keep one digit fixed, the digit supports the leg stably. When encountering different transverse gradients of the slope, the feet show different adaption. When the fixed digit is located on the left part of its workspace, the foot shows greater capacity of absorbing energy (Fig. 4) and the free digit gets more workspace to change its configuration. The energy variation reaches its maximum at the lateral slope angle of 2° (Fig. 5). The foot shows a greater capacity to absorb energy when the fixed digit is on the upper position. Thus, when landing the transverse slope, ruminants need to ensure the upper digit is fixed. Though fastening one digit may enhance stability, it weakens the absorption of the foot and may be very difficult for ruminants to fasten just one digit. If both digits are allowed to change the configurations, the energy variation will be much greater. Let one digit (Branch I) change among 13 configurations, and the energy variation reaches its maximum across level (Fig. 5). Consistent with greater lateral slope angle, the energy variation decreases; landing on the transverse slope will reduce the maximal energy variation and it is best for ruminant to land the feet on a level surface. To absorb more energy and stabilize the feet, ruminants are encouraged to land on the level ground or small gradient ground when running or hopping (great GRF) with symmetric digits.

After discussing the movement of symmetric digits on inclined and hard terrain, the asymmetric digits are taken into account. As Fig. 6 shows, longer lateral digits increase the best lateral slope angle (corresponding to the peak energy variation). If cattle with longer lateral digits are housed or live on the level and hard ground, the ability to absorb energy decreases. The ligaments in the feet may be overtaxed during movement, for articular ligaments that limit the joint rotation and for the interdigital ligaments that limit the detaching of digits. This may be another factor for foot disease or lameness besides the overload of digits (Van der Tol et al., 2003). Raising cattle on a slope disfavored because left limbs and right limbs adapt to the opposite slope angle. Caret-like ground may be one approach to reduce the incidence rate of foot disease.

Nevertheless, when ruminants tread on a softer surface, the grade angle of the surface of the foothold can adapt to the load, i.e. the slope is compressed to access the best lateral angle. Thus, the feet gain greater capacity to absorb energy. This implies that soft ground is conducive to the locomotion of ruminants. It has been shown that the digits of ruminants are well-adapted to the soft ground (Flower et al., 2007; Hernandez-Mendo et al., 2007) and living on hard surfaces leads to the hoof lesions of cattle (Sogstad et al., 2005; Somers et al., 2003).

Another remarkable finding is that the relative length change within one digit does not exert influence on the energy storage. Once the overall length is determined, the ability of energy storage is ascertained. Hence, we may focus more on the asymmetry of the overall length of digits, rather than the proportion of phalanges.

### Conclusion

This paper builds an equivalent kinematic model of the feet of ruminants, which decouples the flexion and extension and lateral movement of feet. The distal part of the foot performs the lateral movement, while the proximal part contributes to the flexion and extension. It reveals one of the motion patterns of ruminants' feet, while robots can be inspired from the foot design. Also, the interaction of two digits and the functions of the distal interdigital ligament based on linear spring hypothesis are analyzed. Level and soft surfaces seem better for ruminants with symmetric digits, even though the feet can adapt to a slope. Asymmetric digits are not supposed to adapt to flat and hard surfaces, and soft surfaces may be the best choice for raising. The investigation provides the energy point of view into how interdigital ligaments restrict the lateral movement. The nonlinear anisotropic mechanical behavior of ligaments needs to be considered in future studies.

## MATERIALS AND METHODS

### Structure of ruminants’ feet and the decoupled mechanical model

The skeleton and joints of ruminants' manus and tarsus show the interspecific similarity, and only vary in size, for instance bovine (Muggli et al., 2011), camel (Wang and Xie, 1992) and sheep (Cuming et al., 1978). The structure of ruminants' manus is shown in Fig. 1A (König and Liebich, 2004). The skeleton of the manus is composed of carpal bones, metacarpal bones and phalanges. In ruminants, there exists the third and fourth digit, which are comprised of the three phalanges. The carpal joint is a composite articulation, which performs as a hinge joint. According to the anatomy of the carpal skeleton complemented by many ligaments, the primary movements of the carpal joint are flexion and extension (König and Liebich, 2004). In ruminants the remaining third and fourth metacarpal bones are fused and no movement is contingent (Sisson, 1921). As hinge joints, the two fetlock joints can only flex and extend, while the pastern joints are saddle joints attributing to the concave-convex shape of the joint surfaces, and act mainly as hinge joints (Meng and Xie, 1997). Even so, served as saddle joints and biaxial joints, flexion and extension and a limited range of lateral movements of the pastern joints are allowed (König and Liebich, 2004). The coffin joints are analogous to the pastern joints. The tarsal joint is also a composite joint. The bones and joints of the metatarsus and digits are similar to those corresponding in the forelimb (Sisson, 1921).

Since two digits contact the ground during the stance phase, only the mechanism constituted by two digits, which are essentially the same in the manus and tarsus, will be discussed. In the following, phalanxes and the corresponding joints are defined as foot. Due to the anatomy [modified from Feng (2014)] and the observation of goats' feet (Zhang et al., 2015), an equivalent mechanism is built in Fig. 1A. The base frame, *S*, is attached to the metacarpal bones. The base connects two identical branches: each consists of one revolute joint and two universal joints.

In the joint notation ***ξ****_ij_* (also screw notation), *i* denotes the branch number, *j* is the joint number within the branch. *θ_ij_* ∈ R(i=1,2; j=1,2,…,5) means the magnitude of the joint ***ξ****_ij_* rotation, and *l_ij_* ∈ R(i=1,2; j=1,2,3) indicates the link of the branch or its length.

The flexions and extensions and lateral movements could not be analyzed easily by using the model in Fig. 1A. When holding the rock firmly during stance phase, the foot forms a single loop mechanism, as discussed in Zhang et al. (2015). The moving platform is attached to the ground. With the roll angle *α* (*x* axis, α≠0), the pitch angle *β* (*y* axis), the yaw angle (0) of the moving platform, and reasonable angular range, the angular excursion of ***ξ***_14_ and ***ξ***_24_ generally remains constant during the movement. In addition, it is found that two digits of the feet synchronously flex and extend in most cases and the coffin joint flexes and extends in a small range. Thus, the equivalent mechanism is simplified. Based on the model in Fig. 1A, we add a constraint that the central points of four universal joints (the common point of intersection for the universal joint axes) lie in the same plane and deactivate the joints of ***ξ***_14_ and ***ξ***_24_. That is, the intersection points of (***ξ***_12_, ***ξ***_13_), (***ξ***_14_, ***ξ***_15_), (***ξ***_22_, ***ξ***_23_), and (***ξ***_24_, ***ξ***_25_) are coplanar. Also, the possible difference between the proximal phalanx (*l*_11_=*l*_21_) is ignored. When the two digits contact the ground at the reference configuration in Fig. 1A, the joints (***ξ***_11_, ***ξ***_12_) and (***ξ***_21_, ***ξ***_22_) have to rotate simultaneously, i.e.
(1)
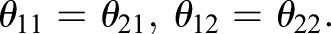
Therefore, the mechanism in Fig. 1A can be simplified as that in Fig. 1B. With roll angle and pitch angle decoupled, the following analysis and discussion are based on the model in Fig. 1B.

The decoupled mechanism can be divided into two independent mechanisms, the upper mechanism and the lower mechanism. The upper mechanism can only turn forward and backward (for ruminants, flexion and extension) for roll, the roll angle *α* is:
(2)
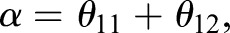
where *θ*_11_= *θ*_21_, *θ*_12_= *θ*_22_.

The lower mechanism performs the lateral rotation (abduction and adduction), and a new base frame is attached to the lower mechanism (Fig. 7). The slope of the line between two points (***p***_1*T*_, ***p***_2*T*_) yields the pitch angle *β* (the lateral angle):
(3)
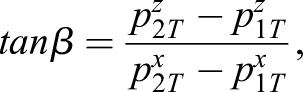
where 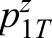 and 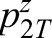 are the *z* components of point ***p***_1*T*_ and ***p***_2*T*_, 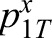 and 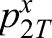 are the *x* components relative to the frame *S* in Fig. 7; but, given the pitch angle, the unique mechanism configuration cannot be obtained, as it is not one-to-one correspoence.

### Kinematics of the lower mechanism

The original pattern of the phalanxes contains five rays. Ruminants have two rays plus two non-functional rays, while horse only has the third ray. There are interdigital ligaments (cruciate ligaments) between the main digits [Fig. 3-46 in König and Liebich (2004)], which are not found in the digit of horse. Distal interdigital ligament bridges the middle phalanxes and distal phalanxes of two digits, while the proximal phalanxes are bound up with each other by proximal interdigital ligament and medial interdigital phalangosersmoidean ligament. Hence, the movement of the digits, which is restricted by the interdigital ligaments, is not independent during stance phase and swing phase. We focus on functions of the distal interdigital ligament in two manners, inextensible ligament and extensible ligament. In order to elaborate on the functions, the distal interdigital ligament is modeled as four conjoint ropes (Fig. 7).

#### Inverse kinematics of the lower mechanism without ligaments

According to screw theory (Yu et al., 2008; Murray et al., 1994), the twist coordinates of the kinematic pair are ***ξ***(***v***,***w***)∈R^6×1^, where ***ω***∈R^3×1^ is the axis of rotation, and ***v***=–***ω***×***q*** (***q***∈R^3×1^ is a point on the axis) if the joint is a revolute joint.

The cross product by ***ω*** is a linear operator, ***ω***×***q*** can be represented using a matrix:
(4)
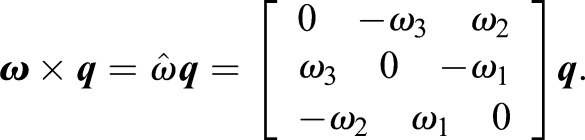
The 4×4 matrix 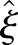 given in Eqn 5 is the generalization of the skew-symmetric matrix 
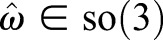

(5)
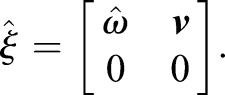
Due to the product of exponentials formula for the manipulator forward kinematic, ***p***_1*T*_ and ***p***_2*T*_ are easily obtained:
(6)
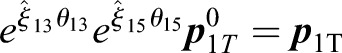

(7)
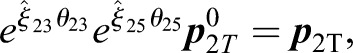
where 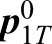 and 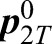 are the initial coordinates at the reference configuration in Fig. 7.

Subtract from both sides (Eqn 6) of a point 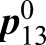 which is at the axis of joint *ξ*_13_ and take the magnitude of both sides:
(8)
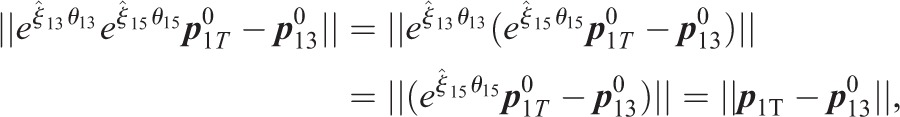
given ***p***_1*T*_ and ***p***_2*T*_, *θ*_13_, *θ*_15_, *θ*_23_, *θ*_25_ are solved by applying the Paden-Kahan subproblem 3 (Murray et al., 1994).

#### The workspace of the lower mechanism without ligaments

Given ***p***_1*T*_, there exist two solutions in general while solving inverse kinematics. The solutions correspond to two possible configurations. If ***p***_15_ is on the left side of line ***p***_13_– ***p***_1*T*_, the configuration of Branch I (in the lower mechanism, the same below) is named left configuration; otherwise, it is right configuration. Then, we need to determine whether the solutions satisfy the constraint conditions. Workspace is considered as a useful measure of the movement range of a mechanism. Two types of kinematic constraints affect the available workspace of the mechanism: joint angle limitations and link interference (Masory and Wang, 1994). The joints of animals cannot rotate 360° because of physical construction. Moreover, since the bones of animals have geometrical shapes and physical dimensions, interference may occur when the mechanism moves. To keep things simple, assume that each link is cylindrical with the same diameter *D*. The shortest distance between two adjacent links should be larger than the diameter *D*. Let *D_i_* be the minimal distance between the centerline of two adjacent links. As the minimal distance between two line segments, *D_i_* may not be equal to the common perpendicular segment of the two adjacent links (Δ*_i_*). If the intersection points of two links with their common normal ** *n_i_*** are known, *D_i_* is equal to Δ*_i_* only if both intersection points are on the links. If one of the intersection points or both are not on the links (i.e. on the extension line), *D_i_* is either the perpendicular distance from an endpoint of one link to the other link or the distance between the endpoints of two links. The detailed method is discussed in Masory and Wang (1994). In conclusion, the inverse solutions of kinematics are subject to the following constraints:
(9)
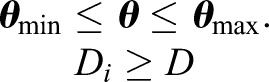
The boundary of the workspace is determined by polar coordinates search method (Masory and Wang, 1994) (from a point within the workspace, the angle *φ* is augmented by Δ*φ* and the radius *ρ* is augmented until the point exceeds the workspace). The area of the reachable workspace is determined by
(10)
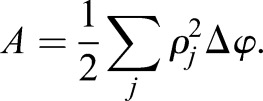


#### Kinematics of the lower mechanism with the inextensible ligament

If the distal interdigital ligament is inextensible and always taut, the elastic ropes become four rigid links articulating two digits. Considering five-bar mechanism at the top, the new mechanism has two DOFs (degree of freedom). Given the angle *θ*_13_ and *θ*_15_, the coordinates of points ***q***_11_, ***q***_12_ and ***p***_15_ can be determined using forward kinematics. The length of virtual links ***q***_11_–***q***_0_, ***q***_12_–***q***_0_, ***q***_21_–***q***_0_, ***q***_22_–***q***_0_ is *l_k_*_1_, *l_k_*_2_, *l_k_*_1_, *l_k_*_2_ (the free length of four elastic ropes in the next section), respectively. As to the triangle ***q***_11_–***q***_0_–***q***_12_, the coordinates of point ***q***_0_ are determined. The problem is to find the cross points of two given circles. Since ***q***_0_, ***p***_23_ and ||***p***_23_–***q***_21_|| are known, ***q***_21_ of the triangle ***p***_23_–***q***_0_–***q***_21_ is solved. Based on cosine theorem, *θ*_23_ can be solved:
(11)
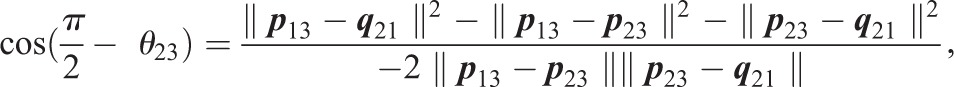
and ***q***_22_ and ***p***_25_ can be determined. *θ*_25_ is solved applying the Paden-Kahan subproblem-rotation about a single axis
(12)
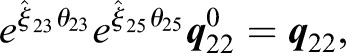
where 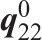 is initial point at the reference configuration (Fig. 7).

#### Kinematics of the lower mechanism with the extensible ligament

If the distal interdigital ligament can stretch, it can be considered as four linear elastic ropes (meeting Hooke's law). If the length of a rope is less than its free length, the elastic force is zero. Thus, the slack ligament imposes no constraints between two digits. The free length of ropes distinguishes between two states of the mechanism: the free state that all ropes exert no elastic forces, and the constrained state that at least two ropes exert the elastic forces.

Given the configuration of Branch I (angular excursion *θ*_13_ and *θ*_15_), the area of the first state of Branch II can be determined by searching the workspace using the above method. The problem corresponds to determining whether four circles have any intersections. The centers are the articulated points of the ropes and the radius is the free length of the elastic ropes. The stiffness of the ropes is shown in Fig. 7. Helly's theorem says if the intersection of every three circles is non-empty, then the four or more circles have a non-empty intersection; therefore it is converted into the three circles intersection problem.

**Fig. 8.**
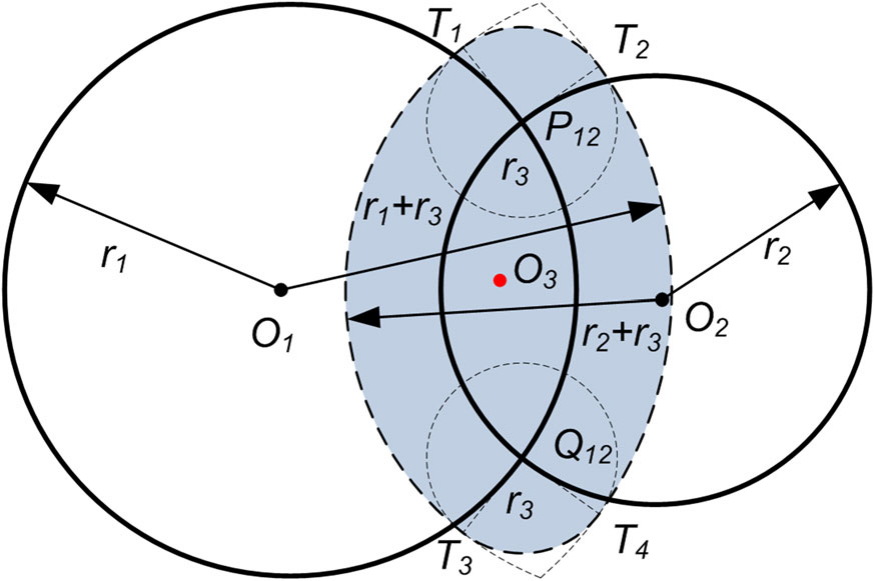
**The three circles intersection problem.** Circle *O*_1_ of radius *r*_1_ and circle *O*_2_ of radius *r*_2_ have two different intersection points *P*_12_, *Q*_12_. The blue area is the intersection of four circles: the one of radius *r*_1_+*r*_3_ centered at *O*_1_, the one of radius *r*_2_+*r*_3_ centered at *O*_2_ and two circles of radius *r*_3_ centered at *P*_12_ and *Q*_12_.

There are, however, some special cases to be discussed. If there is no intersection in two circles, then three circles have no intersection. If two circles are internal circles, the intersection of the smaller circle of the two and the third one is the intersection of the three circles. If one circle is externally tangent to another, we determine if the intersection point is in the third circle. In general, the two circles have two intersection points. If the center of the third circle is within the blue area in Fig. 8, the three circles have a non-empty intersection.

If the mechanism is not in the free state, it enjoys the second state. Given the angles of joints *θ*_13_, *θ*_15_, *θ*_23_ and *θ*_25_, if all the four elastic ropes exert the elastic forces, i.e. the length of all ropes is greater than the free length, the intersection point ***q***_0_ is determined by solving the nonlinear equilibrium equations at the point ***q***_0_:
(13)
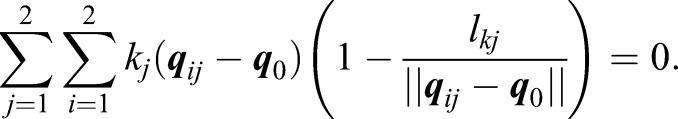
The solutions can be solved using Newton-Euler method, which have to satisfy the following inequalities:
(14)
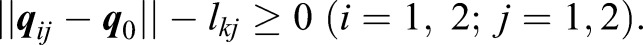
If ***q***_0_ is known, the elastic potential energy of four ropes is determined:
(15)
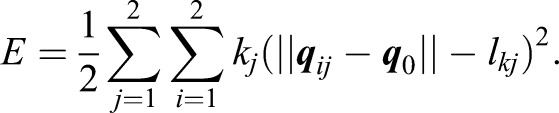
If Eqn 14 is not satisfied, one or two ropes may be relaxed and the corresponding energy should be culled.

### Parameter determination

In this paper, the digits of bovine are chosen as an example. The method above can also be used to analyze other ruminants with the similar structure of feet. Based on the average length of the digits from both the manus and the tarsus (Muggli et al., 2011), the parameters in the mechanism are acquired (Table 1), where the length of the platform between two digits (*l*) is an estimate. For lack of concrete data and analysis, the shortest distance between two adjacent links is assumed to be greater than 27 mm, which is larger than the estimated width of the first phalanx (26 mm).

Previous measurements of goat's feet indicated that the maximum angular excursion of MTP and MCP during stance phase (level, uphill and downhill) was 26.1° (Lee et al., 2008). The maximal extension angle at fetlock joint of cows was about 20° during the middle of stance phase (Carvalho et al., 2007). Another study presented that the maximal range at MCP and MTP of dairy cows was 59.3° (Phillips and Morris, 2001); however, no lateral angular range of digits of ruminants was reported, so the range from reference configuration (***θ***=0) is assumed as shown in Table 1. Meanwhile, the ligament attachments are assumed to lie in the middle of the corresponding phalanx and the length of ropes is the free length at the configuration shown in Fig. 7 (the initial ***q***_0_ has the same *z* coordinate as ***p***_15_ and ***p***_25_). If the ligament is isotropic and has the identical per-length stiffness *K* (100 N), the stiffness of the ropes is determined
(16)
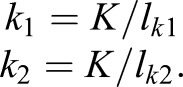
Many reports indicate the differences in the length of the digital bones in cattle (Nuss and Paulus, 2006; Muggli et al., 2011). The cattle have longer lateral digits in both forelimb and hindlimb. The lateral proximal phalanx and middle phalanx are longer than its medial counterparts, while the medial distal phalanx is longer than the lateral one. The relative difference of the proximal phalanx is smaller than other phalanges. We thereupon introduce the asymmetry of middle phalanx and distal phalanx. Moreover, the following discussion suggests that neglect exerts very little influence on the results. Branch II is identified with the lateral digit. To analyze the effect of asymmetry, the length of Branch II is changed whereas Branch I remain the same as the reference value. According to Muggli et al. (2011), the digits meet the conditions:
(17)
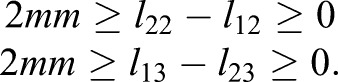


### Data calculation across configurations

The functions of the feet during locomotion are investigated in two ways. First, assume the two digits are symmetric: 13 configurations of Branch I are chosen to assess the impact on the workspace of Branch II. All corresponding endpoints including these of the maximal and minimal *x* coordinates and the minimal *z* coordinate are in a parabola (Fig. 2). Given the constraints (Eqn 9) and the configurations of Branch I, the area of workspace of Branch II can be determined. Based on the result above, we assess the capacity of the feet to perform the lateral angle and reveal the functions of the distal interdigital ligament during stance phase. Given the configurations of Branch I and the lateral angle of the lower mechanism (the slope angle of the line through two endpoints of both Branches), the transverse slope where two branches stand is determined. The endpoint of Branch II can move along the slope, if the slope intersects with the workspace of Branch II. There is no relative movement between the base (Fig. 7) and the slope in this motion (Branch I is fixed). Then the elastic potential energy of the ligament is calculated. The maximal variation of elastic potential energy is determined, implying the capacity of energy absorption
(18)
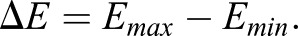
Second, asymmetric digits are introduced to our analysis. Given the workspace boundary and lots of configurations within the workspace of Branch I and Branch II, the lateral slope and the maximal energy storage are determined analogously.

In the process, the multi-solution problem needs to be paid more attention. The left configuration and right configuration of Branch II are calculated respectively, as the configurations cannot be transformed during a continuous motion.
